# In-Depth Characterization and Validation of Human Urine Metabolomes Reveal Novel Metabolic Signatures of Lower Urinary Tract Symptoms

**DOI:** 10.1038/srep30869

**Published:** 2016-08-09

**Authors:** Ling Hao, Tyler Greer, David Page, Yatao Shi, Chad M. Vezina, Jill A. Macoska, Paul C. Marker, Dale E. Bjorling, Wade Bushman, William A. Ricke, Lingjun Li

**Affiliations:** 1 School of Pharmacy, University of Wisconsin-Madison, Madison, WI, 53705, USA; 2 Department of Chemistry, University of Wisconsin-Madison, Madison, WI, 53706, USA; 3 Department of Biostatistics & Medical Informatics, University of Wisconsin-Madison, Madison, WI, 53706, USA; 4 School of Veterinary Medicine, University of Wisconsin-Madison, Madison, WI, 53706, USA; 5 George M. O'Brien Urology research Center, University of Wisconsin-Madison, Madison, WI, USA; 6 Center for Personalized Cancer Therapy, University of Massachusetts, Boston, MA, 02125, USA; 7 Department of Urology, University of Wisconsin-Madison, Madison, WI, 53792, USA

## Abstract

Lower urinary tract symptoms (LUTS) are a range of irritative or obstructive symptoms that commonly afflict aging population. The diagnosis is mostly based on patient-reported symptoms, and current medication often fails to completely eliminate these symptoms. There is a pressing need for objective non-invasive approaches to measure symptoms and understand disease mechanisms. We developed an in-depth workflow combining urine metabolomics analysis and machine learning bioinformatics to characterize metabolic alterations and support objective diagnosis of LUTS. Machine learning feature selection and statistical tests were combined to identify candidate biomarkers, which were statistically validated with leave-one-patient-out cross-validation and absolutely quantified by selected reaction monitoring assay. Receiver operating characteristic analysis showed highly-accurate prediction power of candidate biomarkers to stratify patients into disease or non-diseased categories. The key metabolites and pathways may be possibly correlated with smooth muscle tone changes, increased collagen content, and inflammation, which have been identified as potential contributors to urinary dysfunction in humans and rodents. Periurethral tissue staining revealed a significant increase in collagen content and tissue stiffness in men with LUTS. Together, our study provides the first characterization and validation of LUTS urinary metabolites and pathways to support the future development of a urine-based diagnostic test for LUTS.

Lower urinary tract dysfunction commonly afflicts the aging population and is manifested as a spectrum of symptoms including frequent urination, urgency, weak stream, incomplete bladder emptying, double-voiding and post-void dribbling. Lower urinary tract symptoms (LUTS) have historically been attributed to benign prostatic hyperplasia (BPH) and increased outlet resistance with secondary effects on bladder function^1^,^2^. However, recent studies suggested a multifactorial etiology and pathogenesis of LUTS, including changed smooth muscle tone, decreased detrusor contractility, prostatic inflammation, increase collagen content, and bladder dysfunction^3^,^4^,^5^,^6^,^7^. The diagnosis of LUTS is often based on patient-reported symptoms which do not always mirror objective measurements, and current medical therapies are not effective in all patients. Though specialized uroflowmetry and cystometry tests can be used by specialists to make definitive diagnoses, these techniques are often not available to health care practitioners in community clinics^8^. Under these circumstances, identifying urine biomarkers indicative of LUTS is of great significance to provide molecular targets to understand disease mechanisms and support objective diagnosis of LUTS.

Metabolomics studies the entire small molecule profile in a biological sample and has been extensively used to characterize metabolic changes in disease states. It can reveal actual processes and cellular conditions within the body at the time of sampling, offering more immediate translational benefit than upstream genomics and proteomics^9^,^10^,^11^,^12^,^13^,^14^. Mass spectrometry (MS)-based metabolomics analysis has led the field in disease biomarker discovery, because MS can provide sensitive, accurate and reproducible measurements of metabolites with a wide dynamic range^15^,^16^,^17^. For disorders of the urinary tract, examination of patient urine by MS-based metabolomics techniques is ideally suited to profile urinary small molecules and identify novel disease-specific signatures as candidate biomarkers.

Due to the large dataset generated from MS-based biomarker discovery, sophisticated bioinformatics tools are vital to eliminate systematic bias and interpret clinically important findings. Conventional statistical tests, such as Student's *t*-test, Mann-Whiney test, and ANOVA, are widely used for biomarker discovery and are simple, fast, and easily interpretable. However, *p*-value based univariate statistics could miss important information regarding the correlations of variables in complex biological processes and their association with specific sample classes. Additionally, it is unrealistic to obtain a sample size equaling the number of features (thousands of compounds) in clinical studies, which increases the statistical challenge and the risk of over-fitting the training data.

In recent years, machine learning algorithms have been applied to data analysis for many different biological disciplines^18^,^19^,^20^,^21^. Predictive models can be built using machine learning algorithms to classify samples into specific groups; such models possess great potential for disease diagnosis and therapeutic evaluation. Feature selection can also be conducted to find the subset of features with the highest discriminatory power; this approach has been applied to drug screening and discovery^22^. Machine learning tools have been proven to provide as good or better classification results than other multivariate analysis methods such as principal component analysis (PCA) or clustering analysis^23^. Many machine learning algorithms can be extended to nonlinear cases whereas PCA is based on linearity assumption. In particular, Bayesian classifiers, the random forest (RF) algorithm, and the support vector machine (SVM) algorithm have been successfully applied to interpret data in microarray gene expression, proteomics, and metabolomics studies^18^,^19^,^20^,^24^. Recognizing the characteristics of different bioinformatics tools, often a combination of several tools is better than any single technique.

Despite the widespread application of LC-MS to disease biomarker discovery, few studies have been designed for the aim of diagnosing disease. Challenges that need to be addressed prior to the general clinical applicability of biomarkers include but are not limited to, the consistency and stability of analytical platforms, the efficiency and accuracy of data analysis methods, the evaluation of biomarker sensitivity and specificity, and the validation of biomarker candidates in general population^13^,^20^,^25^,^26^,^27^,^28^. In this study, we established a comprehensive workflow integrating MS-based analytical approaches and advanced bioinformatics tools to address some of these issues, and we then applied this workflow to LUTS biomarker discovery. Recognizing the complex manifestations of LUTS, we have focused on the subpopulation of male patients with urinary frequency and urgency symptoms and hypothesized that urinary metabolomics profiling can generate a panel of biomarkers that are sufficient to classify patients into disease or non-diseased groups.

## Results

**Figure 1:**
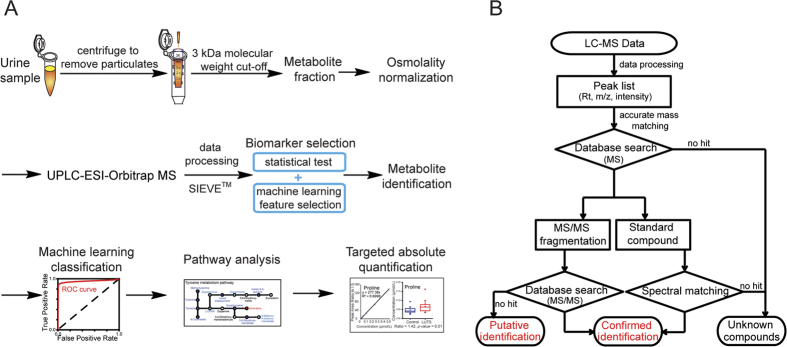
Comprehensive workflow of urinary metabolite biomarker discovery of LUTS (**A**) and flowchart of metabolite identification process (**B**). Accurate mass matching with multiple online databases was conducted with a mass error ∆ppm < 5. Because there are much fewer entries in MS/MS metabolite database compared to MS database, features with matching results in MS but not in MS/MS databases were considered putative identifications.

The workflow for LUTS biomarker discovery is shown in Fig. 1. It integrates urine sample preparation, instrumental analysis, data processing, machine learning feature selection combined with statistical test for biomarker selection, metabolite identification, classification model construction, metabolic pathway analysis, and biomarker verification by absolute quantification.

### Technical Reproducibility of Metabolomics Platform

Quality control (QC) is of utmost importance in large-scale metabolomics biomarker studies to ensure stable system performance and limit experimental bias. A QC standard was prepared as a pooled mixture of aliquots from all urine specimens (26 LUTS patients and 20 control patients). The QC sample was injected before and frequently throughout the analytical run to monitor instrument stability. Technical reproducibility of the platform was assessed by analyzing the QC sample repetitively within the same day and across months. The average mass deviation was less than 1 ppm across months and the average relative standard deviation (RSD) for retention time were 0.9% (intraday) and 5.4% (interday). The average RSD for peak areas were 7.8% (intraday) and 19.6% (interday). LC-MS peak areas are highly correlated between technical replicates of both inter-and intra-day injections (Supplementary Fig. S1). The metabolomics profiling platform yielded consistent peak area, m/z and retention time for reliable comparisons of metabolite profiles between LUTS patients and controls.

### Selection and Identification of Candidate Biomarkers

Overall 2802 aligned spectral features were detected in the LUTS patients vs. control data set. The accurate and efficient selection of candidate biomarkers is achieved by combining machine learning feature selection and traditional statistical test, where 118 features were selected from all 2802 peaks for subsequent metabolite identification.

**Figure 2:**
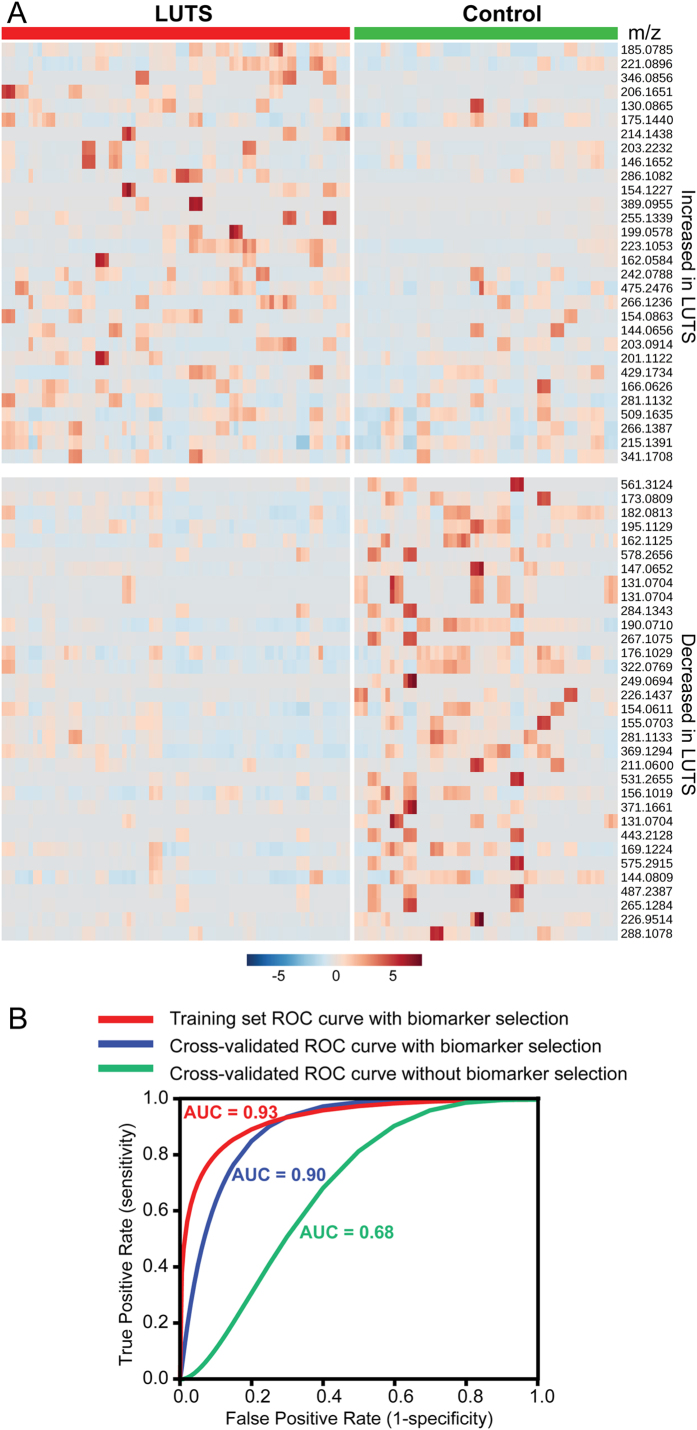
Heat map (**A**) and ROC analysis (**B**) using 63 identified metabolites. The heat map is grouped by disease status and the trend of relative quantification. Shades of red and blue represent metabolite peak areas relative to the median. ROC analysis was carried out to evaluate classification model established with linear SVM algorithm and leave-one-patient-out cross-validation.

Because of the complexity of a metabolome and the absence of a complete metabolite database, metabolite identification is one of the most challenging tasks in metabolomics studies. The use of a high-resolution and accurate-mass (HR/AM) Orbitrap MS and the strict evaluation of QC samples to ensure system reproducibility laid the basis of the successful metabolite identification. Following the designed flowchart in Fig. 1B, a total of 63 metabolites were identified (Supplementary Table S3), and examples of ID confirmation were illustrated in Supplementary Fig. S2. A list of representative metabolites was shown in Table 1. The heat map of the 63 identified metabolites was displayed in Fig. 2A. The blue and red heat map provided a direct visual comparison of relative expression levels of metabolites (rows) grouped by the sample type (columns).

### Binary Classification Model and Statistical Validation

A predictive model for patient classification was constructed using the 63 identified metabolites dataset with the linear SVM algorithm. SVM algorithm is especially robust in handling noisy data and generally not susceptible to outliers, which is well-suited for metabolomics data set^29^. On the training set, this model classified the LUTS vs. control patients with an AUC ROC of 0.93 (Fig. 2B). In order to evaluate whether this model is over-fitting and how it can be expected to perform on future patients, our process of biomarker selection and classification model construction was evaluated: the entire biomarker selection process was repeated using just the training set for each fold, and the resulting features were used to construct the predictive model for that fold, which was then applied to the held-aside test patient for that fold. This cross-validated AUC ROC was 0.90; relatively modest difference from 0.93, indicating that the over-fitting was small. By comparing the results without biomarker selection (AUC ROC of 0.68 for 2802 features), the established model demonstrated significantly increased discriminatory power and prediction accuracy.

### Metabolic Pathway Analysis

**Figure 3:**
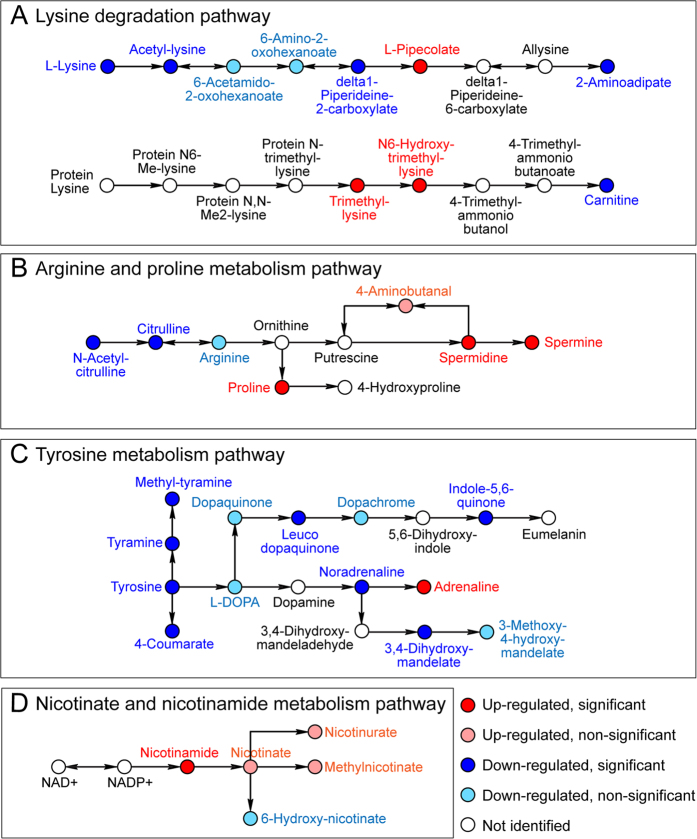
Potentially regulated metabolic pathways. Identified metabolites with direction of changes were indicated with different colored circles. The complete KEGG pathway maps including all the identified metabolites are displayed in Supplementary Fig. S3.

In the human body, metabolites can act synergistically within functionally defined pathways. Metabolic pathway analysis is based on the association between identified metabolites and their related biological processes. Besides the 63 identified candidate biomarkers, an additional 105 metabolites were putatively identified by accurate mass matching (Δppm < 1) in order to include as many metabolites in given pathways regardless of their statistical significance. Eventually, four potentially regulated metabolic pathways were identified, the lysine degradation pathway, the arginine and proline metabolism pathway, the nicotinate and nicotinamide metabolism pathway, and the tyrosine metabolism pathway (Supplementary Table S1). The potentially disrupted metabolic pathways were illustrated in Fig. 3 (important pathway segments) and Supplementary Fig. S3 (complete KEGG maps). Given the complexity of the selected metabolic pathways, it is possible that the entire pathway was altered, or only specific fragments within the pathway were perturbed in the disease state.

### Biomarker Verification by Absolute Quantification

**Figure 4:**
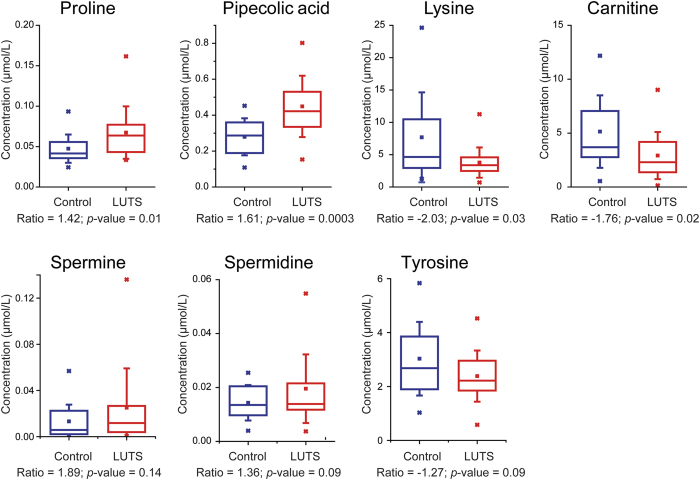
Box plots of absolute quantification of selected metabolites in urine. Each box contains the metabolite concentrations from 26 (LUTS) or 20 (Control) urine samples. Box denotes 25^th^ and 75^th^ percentiles; line within box denotes 50^th^ percentile; whisker denotes standard deviation.

Candidate biomarkers were further verified by absolute quantification using selected reaction monitoring (SRM). Seven metabolites were selected from represented metabolic pathways including proline, pipecolic acid, lysine, carnitine, spermine, spermidine, and tyrosine (Table 2). An eight-point standard curve was constructed for each metabolite with a fixed concentration of the corresponding isotopically labeled internal standard. Excellent linearity (average R^2^ = 0.9986) was achieved for each metabolite across three orders of magnitude in dynamic range (Supplementary Fig. S4). Following this assessment of dynamic range, we performed targeted absolute quantification of the seven metabolites in 46 clinical urine samples. As illustrated in Fig. 4, all of the metabolites exhibited consistent changing trend between absolute and relative quantification, validating our quantitation method and producing important molecular targets for future mechanistic study.

### Collagen Assessment

**Figure 5:**
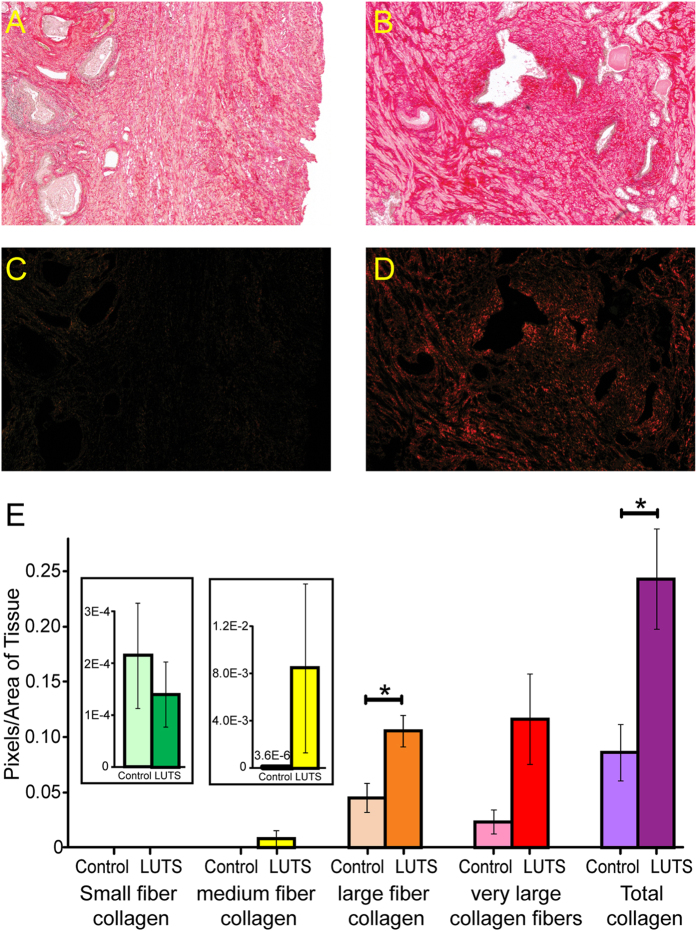
Determination of prostatic collagen content in patients. Picrosirius red stained images of tissues were captured under brightfield illumination (**A**, Control; **B**, LUTS) and under polarized light (**C**, Control; **D**, LUTS). Collagen content was colormetrically quantified for fibers with different sizes and the total collagen (**E**). (Note, *p-value < 0.05).

Lower urinary tract inflammation and fibrosis are very commonly observed in prostatic tissues from male LUTS patients, which has recently been associated with increased symptom severity and risk for clinical progression of LUTS/BPH^3^,^30^,^31^,^32^. But the mechanistic and molecular basis for this association are unclear. Fibrosis is an aberrant wound-healing process downstream of inflammation, which can be characterized by myofibroblast accumulation, collagen deposition, extracellular matrix remodeling and tissue rigidity^5^. Many identified dysregulated metabolites in urine are related to collagen synthesis and deposition, such as metabolites in the arginine and proline metabolism pathway^33^,^34^,^35^. In order to investigate prostatic fibrosis as a potential contributor of LUTS and correlate metabolite dysregulations with changed collagen deposition indicating fibrosis, we performed a follow-up study to assess collagen content and tissue stiffness in periurethral prostatic tissues of LUTS patients vs. controls. The collagen content was determined to be significantly higher in LUTS patients (n = 5) than in control patients (n = 7), as illustrated by Picrosirius red staining images and colorimetrically quantified birefringes (Fig. 5). Total collagen content was significantly (*p*-value = 0.04) increased in men with LUTS and large collagen fibers (orange, *p*-value = 0.02) were significantly increased as well. Median collagen fibers (yellow, *p*-value = 0.37) and very large fibers (red, *p*-value = 0.13) were also increased in LUTS patients but not reaching statistically significance, probably due to limited sample size. The average tangent modulus of periurethral prostatic tissue was 1978 ± 314 kPa for LUTS patients vs. 411 ± 274 kPa for controls (*p*-value = 0.00002), representing significantly greater tissue stiffness in LUTS group. These data suggest that increased collagen content and tissue stiffness in the periurethral prostatic area indicating fibrosis may contribute to the dysregulation of urine metabolites related to collagen synthesis and deposition.

## Discussion

A panel of metabolite biomarker candidates and their related metabolic pathways were successfully generated from our designed workflow. The established binary classification model has great potential for future development of a urine-based diagnostic test of LUTS. In addition, the key metabolites and their related bioprocesses are discussed here to help elucidate the underlying molecular functions involved in LUTS development and progression; particularly their possible associations with the function of lower urinary tract which involves complex regulation of smooth muscle contraction and relaxation and also the coordination of neural networks.

The arginine and proline metabolism pathway was found to be distinctly perturbed with more than 30 identified metabolites (Fig. 3B and Supplementary Fig. S3B). This pathway has been known to be related to the synthesis of collagen^33^,^34^,^35^,^36^. Two crucial polyamines, spermine and its precursor spermidine were significantly increased in LUTS patients’ urine and verified by absolute quantification. Prostatic tissue is one of the highest polyamine producing organs in the body^37^. Studies suggested that polyamines can promote collagen production and cell proliferation. Arginase activity can also have direct effects on fibrosis, which is a potential contributor to LUTS/BPH^3^,^36^,^38^. The increased collagen content and extracellular proteins causes tissue stiffness as well as reduced tissue elasticity and compliance^5^. Additionally, spermidine regulates Ca^2+^ influx and Na^+^, K^+^ ATPase activity, which is closely related to the contraction activity in the detrusor of urinary tract and the bladder smooth muscle^39^. Given that we and others have identified that the accumulation of extracellular matrix^3^,^5^, especially collagen^31^, is associated with LUTS in men, the identification of collagen precursors found within the urine is suggestive that these metabolites are putative biomarkers of LUTS. Perhaps more importantly, these putative biomarkers may be informative to personalized medical therapy treatment in men presenting with LUTS as current therapies do not target the extracellular matrix and hence may not be effective in men presenting with these urinary markers.

Methylated intermediates in the lysine degradation pathway were found to be significantly increased in LUTS patient urine, including dimethyllysine, trimethyllysine, and hydroxy-trimethyllysine (Fig. 3A). Methylation patterns of lysine serve as important biological signals which establish chromatin structure and regulate carnitine biosynthesis and fatty acid oxidation^40^,^41^. It was reported that the methylation of histone H3 at lysine 4 (H3-K4) is associated with transcriptional regulation of the prostate-specific antigen (PSA) gene in the prostate cancer cell line^42^. But further targeted investigation into methylated metabolites is necessary.

Tyrosine metabolism is related to signal transduction in human body, and tyrosine kinase can modulate smooth muscle contraction through Ca^2+^ sensitization^43^. Majority of the identified metabolites in tyrosine metabolism were down-regulated in LUTS patients (Fig. 3C). Results of pathway analysis also indicated potential disruption of the nicotinate and nicotinamide metabolism pathway in LUTS (Fig. 3D), in which NADP+ and NAD+ are important cofactors for energy metabolism such as glycolysis and fatty acid catabolism. Nicotinamide, significantly elevated in the LUTS patient, was also found to associate with the regulation of inflammatory actions which is one of the most important etiologies of LUTS^4^,^44^.

These possible metabolic correlations with disease etiologies are consistent with our recent urinary proteomics study of LUTS in men, which identified and relatively quantified a group of proteins related to fibrosis and inflammatory responses^45^. Changes in smooth muscle tone, prostatic hyperplasia, inflammation, and increased collagen content have been identified in urology studies as possible contributors to urinary dysfunction^3^,^4^,^7^,^31^,^44^. However, in order to confirm the possible correlation between the changes of metabolites and functional bioprocesses of LUTS, future targeted mechanistic studies are necessary via cell culture or justified mouse models of LUTS^4^,^15^,^46^,^47^,^48^,^49^,^50^.

In summary, we have developed and implemented an in-depth metabolomics analytical platform combining MS-based analysis and advanced machine learning bioinformatics tools. The established method was successfully applied to study LUTS in men, resulting in important disease-associated biomarker and pathway candidates as well as a sensitive and specific classification model for potential non-invasive diagnosis of LUTS. The hypothesized metabolic correlation with collagen deposition was further studied in periurethral prostatic collagen staining. Aging female patients are also known to develop LUTS symptoms. Unlike LUTS in male which has historically been attributed to benign prostatic hyperplasia, LUTS in female is more likely associated with other factors such as bladder dysfunction, urinary tract infection, and postmenopausal urogenital changes. Because of the different etiology of LUTS in female^51^,^52^, we only focused on LUTS in male in the present study. The established workflow can also be applied to future studies of LUTS in female. It is also worth pointing out that the metabolites and pathways generated in this study are only candidate signatures of LUTS. Future targeted mechanistic study and clinical validation with a separate large cohort of patient samples are necessary before the real usage in clinical practice. Together, this study provided a well-designed methodology and promising molecular targets that are useful for future clinical diagnosis and pathophysiological study of disease.

## Methods

### Clinical Sample Collection

This study was approved by Institutional Review Board (IRB) Protocol and conducted under the guidance of the University of Wisconsin-Madison Human Research Protection Program (HRPP). All human subjects provided informed consent before participating in this study. Midstream urine samples were collected from 26 patients with LUTS and 20 controls without LUTS in the Urology clinic of the University of Wisconsin Hospital according to the approved IRB. Because of the physiological and anatomic differences of lower urinary tract between female and male, the etiology and risk factors of developing LUTS are often separately studied for female and male patients^51^,^53^. In this study, the recruited LUTS patients were men with significant urinary frequency and urgency for a duration of more than 6 months as described by the American Urological Association Symptom Index (AUASI) frequency + urgency symptom scores of >7^8^. Control male patients had no history of significant LUTS and the symptom score ≤3 (detailed patient inclusion and exclusion criteria are provided in Supplementary Fig. S5). Because many LUTS patients have a history of other urologic conditions, including renal cell carcinoma, renal cystic disease, kidney stones, erectile dysfunction, hydrocele, and low-grade prostate cancer, controls were also selected from patients with such diagnostic history not specifically associated with LUTS in order to provide a spectrum of patients that can dilute the effect of confounding variables. The age and body mass index were matched between recruited LUTS and control patients (Supplementary Table S2). After collection, all midstream urine samples were centrifuged at 1000 g for 10 min, spiked with sodium azide, de-identified, and stored at −80 °C until analysis.

In order to compare collagen levels in men with or without LUTS, human periurethral prostatic tissues were collected from a separate group of LUTS patients (n = 5) and age-matched controls (n = 7) assessed under AUASI criteria. Periurethral tissues were procured at surgery from men undergoing radical prostatectomy, who had completed the AUASI within 30 days before surgery. Patient clinical information was provided in Supplementary Table S2. Experimental details were described previously^3^,^31^. Tissue samples and related clinical information were obtained with IRB approval.

### Urine Sample Preparation

Urinary metabolites were separated from large molecules using 3 kDa molecular weight cut-off (MWCO) ultracentrifugation filters (Millipore Amicon Ultra, MA) according to the manufacturer’s protocol. The flow-through fractions were collected as urinary metabolites. Osmolality of each metabolite fraction was measured by a freezing-point depression osmometer (Osmometer Model 3250, Advanced Instruments, MA) and metabolite samples were diluted to achieve the same osmolality. By pre-acquisition normalization of the urine, we ensured that each sample has the same osmolality and similar total metabolite concentration before instrumental analysis (Supplementary Fig. S6). For absolute quantification, a mixture of isotopically labeled internal standard (I.S.) was spiked in urine samples before instrumental analysis.

### LC-MS and LC-MS/MS Analysis

Ultra-performance LC-MS analyses of urine samples were conducted using a Dionex UltiMate 3000 LC system coupled with a Q-Exactive^TM^ Orbitrap mass spectrometer (San Jose, CA). Urinary metabolites were separated with a 20 min gradient on a Phenomenex biphenyl column (2.1 × 100 mm, 2.6 μm, 100 Å) at a flow rate of 0.3 ml/min. Mobile phase A was 0.1% formic acid in H_2_O and mobile phase B was 0.1% formic acid in MeOH. The gradient was set as follows: 0–5 min, 0–3% solvent B; 5–15 min, 3–40% solvent B; 15–18 min, 80% solvent B. Full MS acquisition scanned from 70 to 1000 *m/z* at a resolution of 70 K. Automatic gain control (AGC) target was 1 × 10^6^ and maximum injection time (IT) was 100 ms. UPLC targeted-MS/MS analyses were acquired at a resolution of 35 K with AGC target of 5 × 10^5^, maximum IT of 50 ms, and isolation window of 2 *m/z*. Collision energy was optimized for each target with higher-energy collisional dissociation (HCD) fragmentation. The injection order of urine samples with 3 technical replicates was randomized to reduce the experimental bias.

### Data Processing and Statistical Analysis

Data files acquired by Thermo Scientific Xcalibur software were processed by commercial SIEVE^TM^ software for peak alignment and framing. Total ion current (TIC) normalization embedded in SIEVE was performed to reduce instrumental variation before directing to statistical analysis. A total of 2802 aligned spectral features were detected after filtering out irreproducible peaks that were present in fewer than three biological replicates from each group (LUTS or control). In order to select mass features whose peak areas differentiate between disease and control group, a Student’s *t*-test was conducted to generate the average fold change and *p*-value of each detected feature. False discovery rate (FDR) correction was used to estimate the chance of false positives and correct for multiple hypothesis testing. The distribution of *p*-values was used to calculate *q*-values using the Benjamin-Hochberg algorithm in R package^54^. Features with both *p*-value and *q*-value < 0.05 were considered statistically significant. For metabolic pathway analysis, the pathway’s *p*-value was calculated as the median of *p*-values of all the identified metabolites involved in the specific pathway. For absolute quantification and collagen staining experiments, the two-tailed Student’s *t*-test was conducted and *p*-value < 0.05 was considered statistically significant.

### Machine Learning Feature Selection

Chromatographic peak areas of detected features generated from SIEVE was input into WEKA 3.6 software^55^ for machine learning based feature selection. Support vector machine (SVM) based attribute evaluation and information gain (IG) based attribute filtering were used to conduct feature selection and rank features based on their contributions to separate LUTS versus control groups. SVM constructs a hyperplane with the maximum margin to separate two groups as widely as possible^29^. IG measures the effectiveness of an attribute in classifying the data based on the entropy measure in information theory^56^. After obtaining the rank of all detected features, the top 100 features in SVM and IG evaluation were selected.

### Metabolite Identification

Significantly altered features from statistical test (both *p-*value and *q-*value < 0.05) and feature selection (top 100 ranking features from both SVM and IG algorithms) were overlapped to compile a list of most significant features. Metabolite identification was performed according to our designed flowchart in Fig. 1B. First, accurate masses of selected features were searched against multiple databases (mass error < 5 ppm) using the MetaboSearch software^57^, including Human Metabolome Database, Madison Metabolomics Consortium Database, Metlin, and LIPID MAPS. Features with matching results from the databases were subjected to LC targeted-MS/MS analysis, and their MS/MS fragments were searched using the MetFrag software^58^ to confirm identities. Metabolite IDs were also confirmed with available metabolite standard compounds.

### Machine Learning Classification

Chromatographic peak areas of identified metabolites were directed into WEKA software to build binary classification models with the linear SVM algorithm, which has been shown to work well in high dimensional data. Leave-one-patient-out cross-validation was carried out to evaluate classification accuracy and measure the proportion of patient subjects correctly classified in this task. This procedure withholds one patient at a time as a test set and uses the rest of the data as a training set and repeats this process until all patients have been used exactly once as the test set and classified. The resulting probability values of LUTS for each patient, when that patient is used for testing, can be used to compute a ROC curve. To test the value of the biomarker selection, a ROC curve was also generated using all the features in the dataset for comparison.

### Metabolic Pathway Analysis

For metabolic pathway analysis, the identities of candidate biomarkers were input into the MetaboAnalyst 2.0 software^59^ to query against the Kyoto Encyclopedia of Genes and Genomes (KEGG) database and generate a list of involved pathways^60^. Only pathways with more than two identified compounds were considered and additional metabolites were identified in order to maximize the metabolite coverage associated with a given pathway, regardless of their *p*-value and feature ranker.

### Collagen Staining

Human periurethral prostatic tissues were subjected to mechanical testing to assess rigidity and stiffness as described previously^3^. The tangent modulus of a tissue sample was measured as the terminal slope of the nominal stress vs nominal strain response in kPa, representing passive tissue stiffness. For collagen staining, human periurethral prostatic tissues were fixed in 10% neutral buffered formalin, embedded in paraffin, and sectioned onto positively charged microscope slides. The tissue sections were stained with Picrosirius red and images were acquired under polarized light as described previously^31^. The different collagen fiber sizes were assessed by imaging and quantitating different colors of birefringence using Image J software suite. Student’s t-test was performed for each color (green, yellow, orange, and red) and total birefringence.

## Additional Information

**How to cite this article**: Hao, L. *et al*. In-Depth Characterization and Validation of Human Urine Metabolomes Reveal Novel Metabolic Signatures of Lower Urinary Tract Symptoms. *Sci. Rep.* **6**, 30869; doi: 10.1038/srep30869 (2016).

## Supplementary Material

Supplementary Information

## Figures and Tables

**Table 1:** Representative candidate metabolite biomarkers of LUTS.

Metabolite	Pathway	KEGG ID	HMDB ID	m/z	∆ppm^c^	Time	Ratio^d^	p-value^e^	q-value
N6,N6-Dimethyl-lysine^a^	Lysine degradation	C05545	HMDB13287	175.1440	0.16	0.76	1.25	0.023	0.025
N-Acetyl-glutamate^a^	Arginine and proline metabolism	C00624	HMDB00341	190.0710	0.12	1.75	−1.59	<0.001	<0.001
Tyrosine^a^	Tyrosine metabolism	C00082	HMDB00158	182.0813	0.59	1.56	−1.41	<0.001	0.001
Spermidine^a^	Arginine and proline metabolism	C00315	HMDB01257	146.1652	0.02	0.64	1.62	0.009	0.016
Carnitine^a^	Lysine degradation	C00318	HMDB00062	162.1125	0.04	0.90	−2.40	<0.001	<0.001
Spermine^a^	Arginine and proline metabolism	C00750	HMDB01256	203.2232	0.76	0.67	2.47	0.007	0.014
Citrulline^a^	Arginine and proline metabolism	C00327	HMDB00904	176.1029	0.05	0.92	−1.69	<0.001	<0.001
2-Octenedioic acid^b^	Unsaturated fatty acid	NA	HMDB00341	173.0809	0.24	12.67	−1.62	0.003	0.009
6-Hydroxypseudooxynicotine^b^	Nicotinate and nicotinamide metabolism	C01297	NA	195.1129	0.52	12.48	−1.57	0.006	<0.001
Pipecolic acid^a^	Lysine degradation	C00408	HMDB00070	130.0865	1.72	1.17	2.02	0.010	0.016

^a^Metabolite ID was confirmed with standard compound.

^b^Metabolite ID was confirmed with MS/MS fragmentation.

^c^∆ppm mass error = 1 × 10^6^ × |detected m/z – theoretical m/z|/theoretical m/z.

^d^Ratio > 0 (positive value) represents up-regulated metabolite; Ratio < 0 (negative value) represents down-regulated metabolite.

^e^*P*-value is calculated using two-tailed Student’s t-test.

**Table 2:** SRM absolute quantification of selected metabolites and their stable isotope-labeled internal standards.

Compounds	m/z → MS/MS	Time	CE^a^	Ratio^b^	p-value^c^	Pathway
Proline	116.0710 → 70.07	0.90	35	1.42	0.01	Arginine and proline metabolism
Proline-D3 (I.S.)	119.0896 → 73.08	0.90	35			
Pipecolic acid	130.0865 → 84.08	1.72	30	1.61	0.0003	Lysine degradation
Pipecolic acid-D9 (I.S.)	139.1428 → 93.14	1.72	30			
Lysine	147.1128 → 84.08	0.68	30	−2.03	0.03	Lysine degradation
Lysine-D4 (I.S.)	151.1379 → 88.11	0.68	30			
Carnitine	162.1125 → 60.08	0.90	45	−1.76	0.02	Lysine degradation
Carnitine-D9 (I.S.)	171.1688 → 69.14	0.88	45			
Spermine	203.2230 → 129.1	0.67	30	1.89	0.14	Arginine and proline metabolism
Spermine-D8 (I.S.)	211.2731 → 137.2	0.67	30			
Spermidine	146.1652 → 72.08	0.64	30	1.36	0.09	Arginine and proline metabolism
Spermidine-D8 (I.S.)	154.2153 → 80.13	0.64	30			
Tyrosine	182.0813 → 136.1	1.56	25	−1.27	0.09	Tyrosine metabolism
Tyrosine-D4 (I.S.)	186.1062 → 140.1	1.54	25			

^a^Optimized collision energy to obtain the highest MS/MS target ion for quantification.

^b^Ratio > 0 : up-regulated metabolite; Ratio < 0 : down-regulated metabolite.

^c^*P*-value is calculated using two-tailed Student’s t-test.
