# Supplementary material for: In-Depth Characterization and Validation of Human Urine Metabolomes Reveal Novel Metabolic Signatures of Lower Urinary Tract Symptoms
Source: Sci Rep. 2016 Aug 9;6:30869. doi: 10.1038/srep30869 (PMC4977550; doi:10.1038/srep30869)
Supplement: Supplementary Information [file srep30869-s1.pdf]

In-Depth Characterization and Validation of Human Urine Metabolomes Reveal Novel Metabolic Signatures of Lower Urinary Tract Symptoms

Ling Hao[1], Tyler Greer[2], David Page[3], Yatao Shi[1], Chad M. Vezina[4,7], Jill A. Macoska[5,7], Paul C. Marker[1,7],Dale E. Bjorling[4,7], Wade Bushman[6,7,#], William A. Ricke[6,7,#], and Lingjun Li[1,2]*

[1]School of Pharmacy, University of Wisconsin-Madison, Madison, WI, 53705, USA, [2]Department of Chemistry, University of Wisconsin-Madison, Madison, WI, 53706, USA, [3]Department of Biostatistics & Medical Informatics, University of Wisconsin-Madison, Madison, WI, 53706, USA, [4]School of Veterinary Medicine, University of Wisconsin-Madison, Madison, WI, 53706, USA, [5]Center for Personalized Cancer Therapy, University of Massachusetts, Boston, MA, 02125, USA, [6]Department of Urology, University of Wisconsin-Madison, Madison, WI, 53792, USA, and [7]George M. O'Brien Urology research Center, University of Wisconsin-Madison, Madison, WI, USA.

[#] These authors contributed equally.

*Corresponding author

Tel.: +1 (608) 265-8491

Fax: +1 (608) 262-5345

E-mail: lingjun.li@wisc.edu

SUPPLEMENTARY INFORMATION

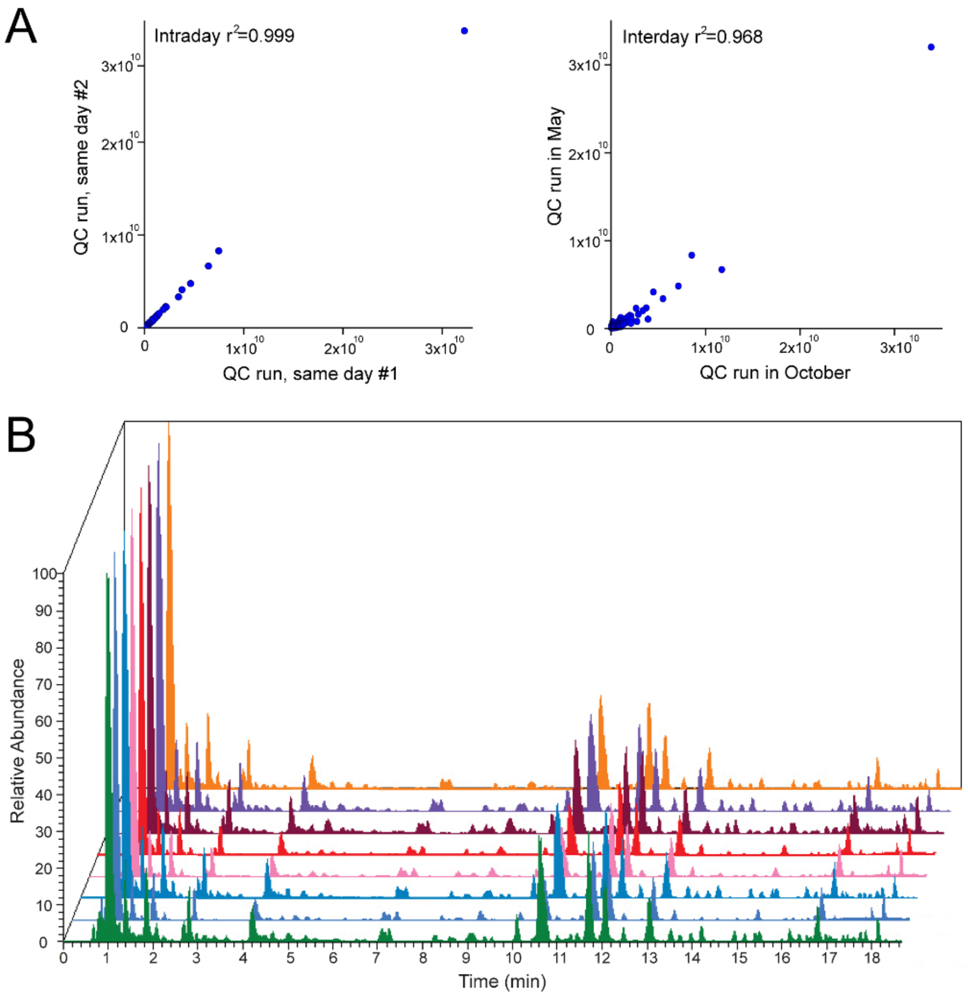

Figure S1. Validation of technical reproducibility of the established metabolomics platform using QC samples. A: Correlation of peak areas between technical replicates for both inter-day and intra-day LC-MS injections. Each data point corresponds to a detected feature across two QC runs within a day or between days. The data point at the upper-right corner of each graph represents creatinine peak area, which is the highest abundance compound in urine. **B**: Base peak chromatograms of eight QC samples injected throughout months.

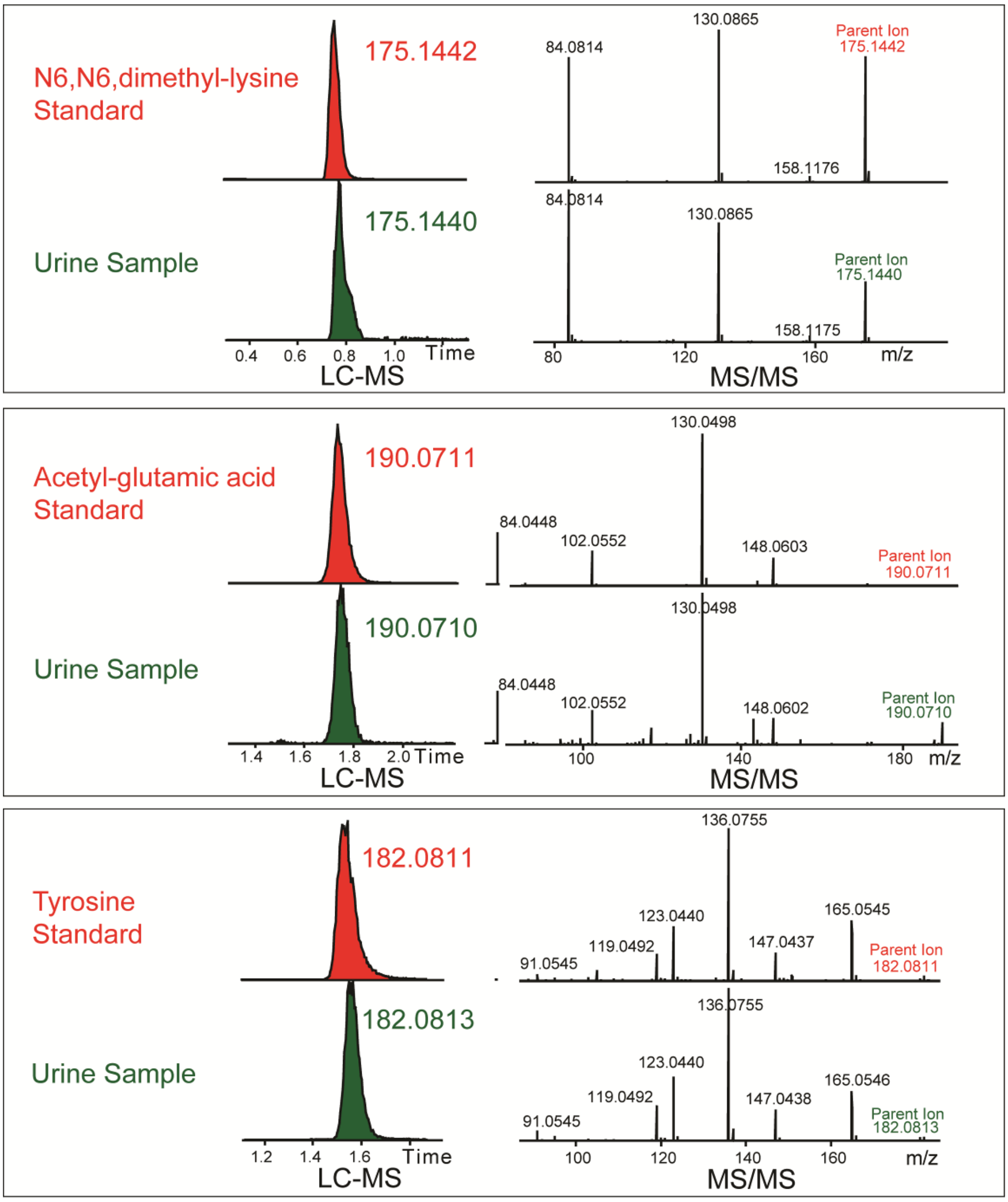

Figure S2. Examples of ID confirmation for metabolite identification. Detected metabolite in urine (green) were confirmed with available standard compounds (red) via the comparison of LC-MS retention time, accurate mass, and MS/MS fragmentation.

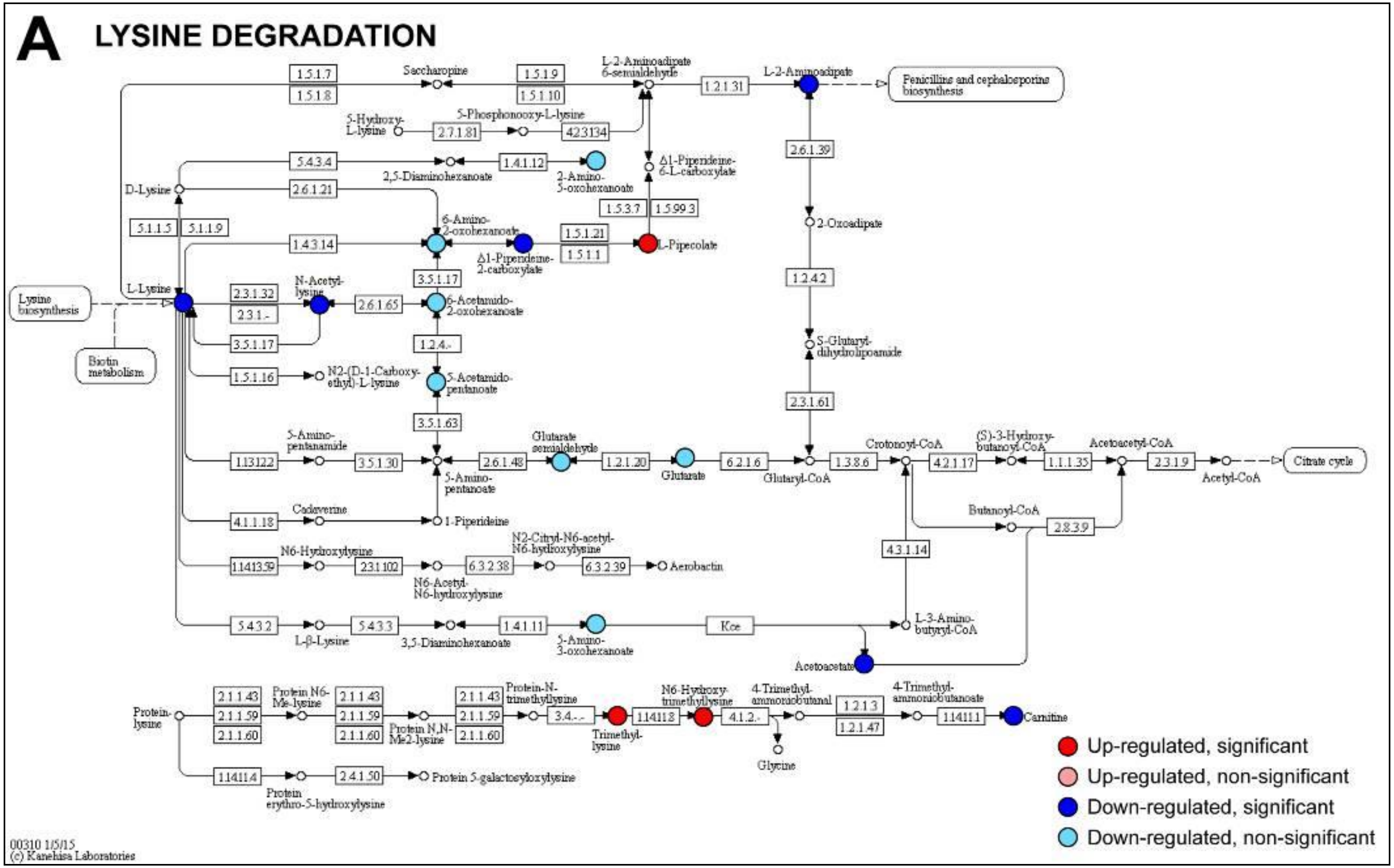
A
LYSINE DEGRADATION
Saccharopine
L-2-Aminoadipate 6-semialdehyde
L-2-Aminoadipate
Penicillins and cephalosporins biosynthesis
1.5.1.7
1.5.1.8
1.5.1.9
1.5.1.10
1.2.1.31
5-Hydroxy-L-lysine
5-Phosphonooxy-L-lysine
2.7.1.81
4.2.3.134
5.4.3.4
1.4.1.12
2,5-Diaminohexanoate
2-Amino-5-oxohexanoate
Δ1-Piperideine-6-L-carboxylate
2.6.1.39
D-Lysine
2.6.1.21
1.5.3.7
1.5.99.3
2-Oxoadipate
5.1.1.5
5.1.1.9
6-Amino-2-oxohexanoate
1.4.3.14
1.5.1.21
1.5.1.1
L-Pipecolate
Δ1-Piperideine-2-carboxylate
3.5.1.17
1.2.4.2
Lysine biosynthesis
L-Lysine
2.3.1.32
2.3.1.-
N-Acetyl-lysine
2.6.1.65
6-Acetamido-2-oxohexanoate
S-Glutaryl-dihydrolipoamide
Biotin metabolism
3.5.1.17
1.2.4.-
1.5.1.16
N2-(D-1-Carboxyethyl)-L-lysine
5-Acetamido-pentanoate
2.3.1.61
3.5.1.63
5-Amino-pentanamide
Glutarate semialdehyde
Crotonoyl-CoA
(S)-3-Hydroxy-butanoyl-CoA
Acetoacetyl-CoA
1.13.12.2
3.5.1.30
2.6.1.48
1.2.1.20
Glutarate
6.2.1.6
Glutaryl-CoA
1.3.8.6
4.2.1.17
1.1.1.35
2.3.1.9
Acetyl-CoA
Citrate cycle
5-Amino-pentanoate
Cadaverine
4.1.1.18
1-Piperideine
Butanoyl-CoA
2.8.3.9
N6-Hydroxylysine
N2-Citryl-N6-acetyl-N6-hydroxylysine
1.14.13.59
2.3.1.102
6.3.2.38
6.3.2.39
Aerobactin
4.3.1.14
N6-Acetyl-N6-hydroxylysine
5.4.3.2
5.4.3.3
1.4.1.11
Kce
L-3-Amino-butyryl-CoA
L-β-Lysine
3,5-Diaminohexanoate
5-Amino-3-oxohexanoate
Acetoacetate
Protein-lysine
2.1.1.43
2.1.1.59
2.1.1.60
Protein N6-Me-lysine
Protein N,N-Me2-lysine
Protein-N-trimethyllysine
3.4.-.-
Trimethyl-lysine
1.14.11.8
N6-Hydroxy-trimethyllysine
4.1.2.-
Glycine
4-Trimethyl-ammoniobutanal
1.2.1.3
1.2.1.47
4-Trimethyl-ammoniobutanoate
1.14.11.1
Carnitine
1.14.11.4
Protein erythro-5-hydroxylysine
2.4.1.50
Protein 5-galactosyloxylysine
Up-regulated, significant
Up-regulated, non-significant
Down-regulated, significant
Down-regulated, non-significant
00310 1/5/15
(c) Kanehisa Laboratories

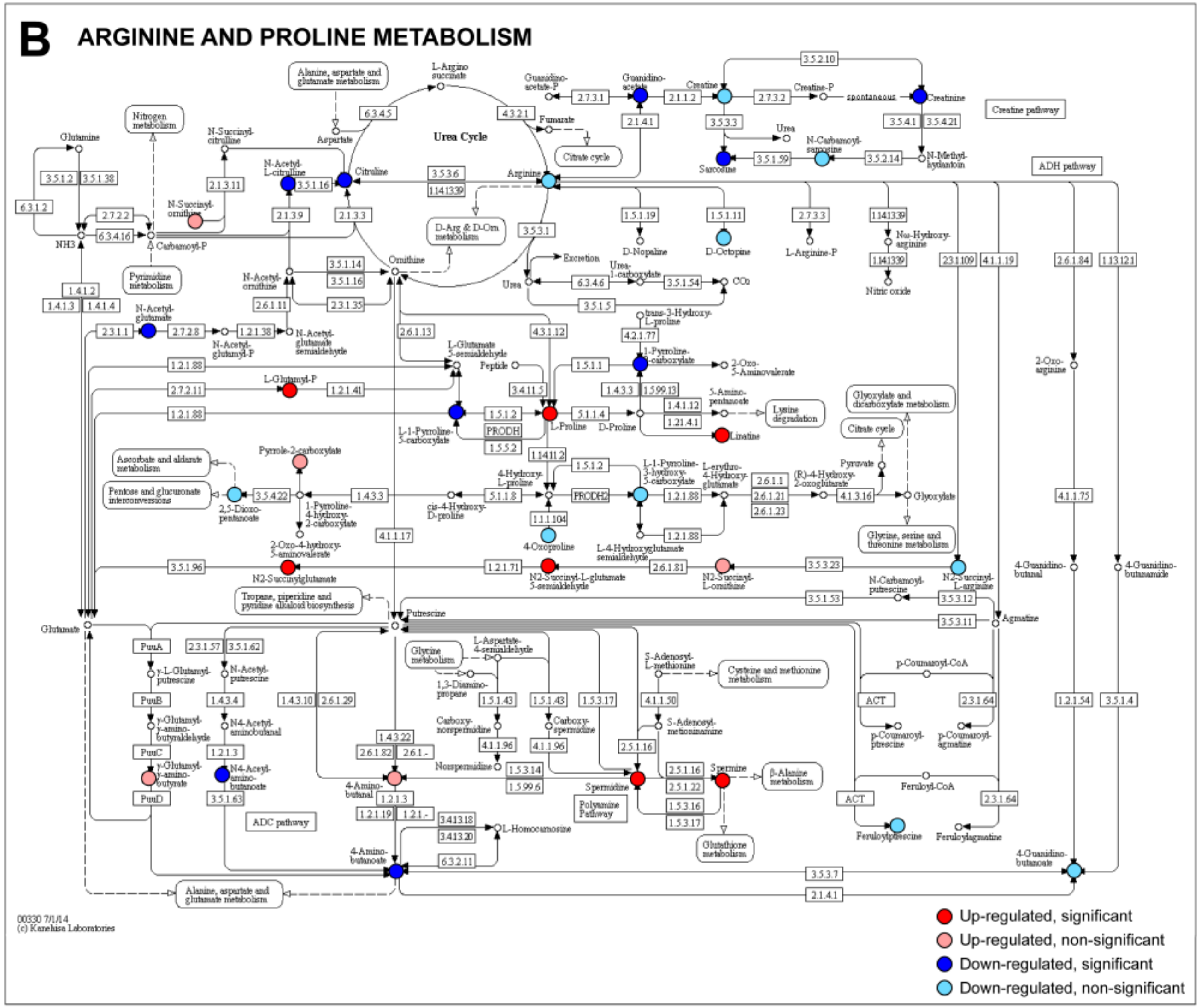
B
ARGININE AND PROLINE METABOLISM
Urea Cycle
Creatine pathway
ADH pathway
ADC pathway
Arginine
Ornithine
Citrulline
Creatine
Creatinine
Sarcosine
Glutamine
Glutamate
NH3
L-Proline
Putrescine
Spermidine
Spermine
Agmatine
Urea
00330 7/1/14
(c) Kanehisa Laboratories
Up-regulated, significant
Up-regulated, non-significant
Down-regulated, significant
Down-regulated, non-significant

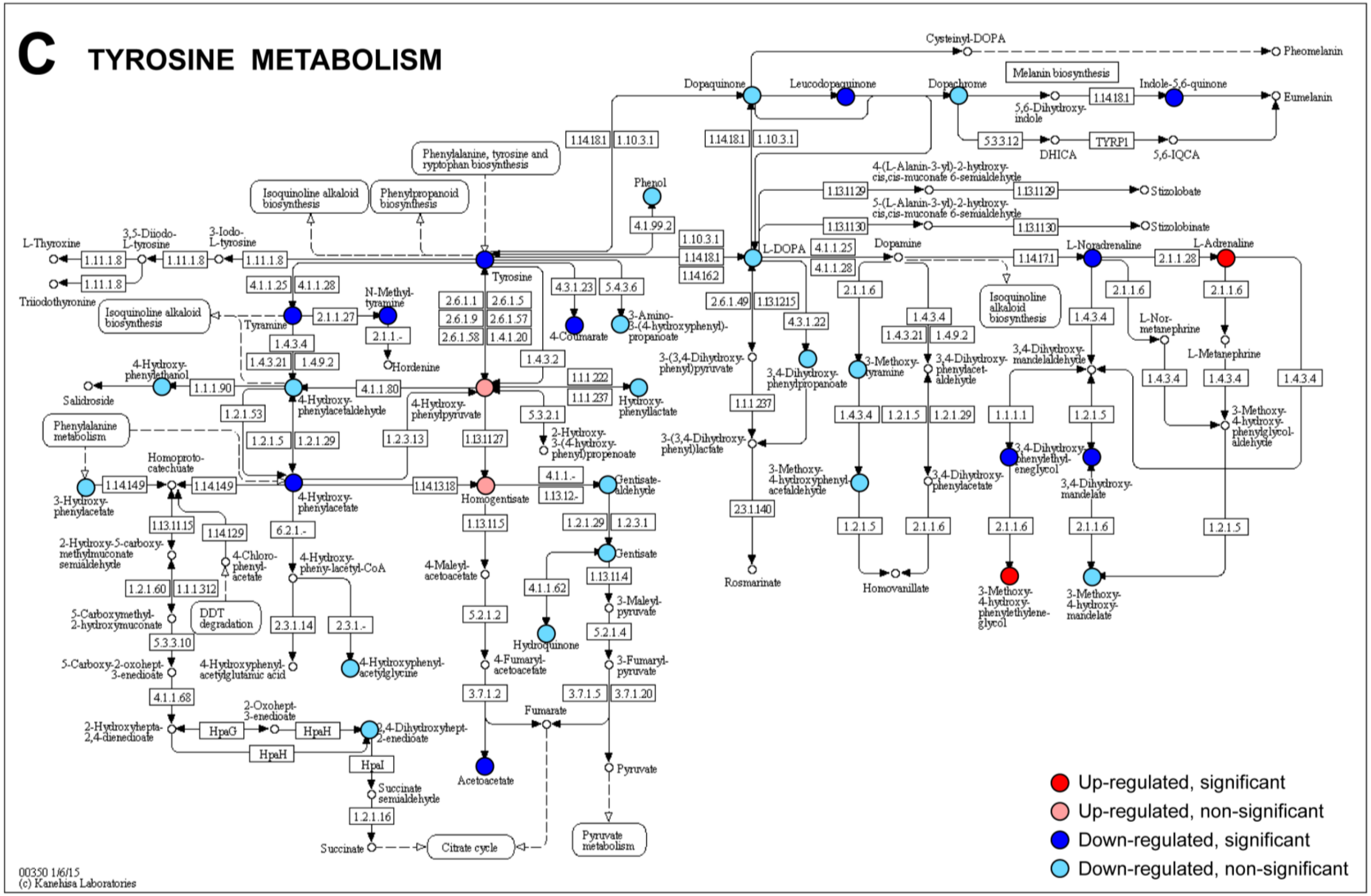
C TYROSINE METABOLISM
Cysteinyl-DOPA
Pheomelanin
Melanin biosynthesis
Dopaquinone
Leucodopaquinone
Dopachrome
Indole-5,6-quinone
Eumelanin
5,6-Dihydroxy-indole
DHICA
5,6-IQCA
TYRP1
Phenylalanine, tyrosine and tryptophan biosynthesis
Isoquinoline alkaloid biosynthesis
Phenylpropanoid biosynthesis
Phenol
4-(L-Alanin-3-yl)-2-hydroxy-cis,cis-muconate 6-semialdehyde
5-(L-Alanin-3-yl)-2-hydroxy-cis,cis-muconate 6-semialdehyde
Stizolobate
Stizolobinate
L-Thyroxine
3,5-Diiodo-L-tyrosine
3-Iodo-L-tyrosine
Triiodothyronine
Tyrosine
L-DOPA
Dopamine
L-Noradrenaline
L-Adrenaline
N-Methyl-tyramine
Tyramine
Hordenine
4-Coumarate
3-Amino-3-(4-hydroxyphenyl)-propanoate
L-Normetanephrine
L-Metanephrine
3-(3,4-Dihydroxy-phenyl)pyruvate
3,4-Dihydroxy-phenylpropanoate
3-Methoxy-tyramine
3,4-Dihydroxy-phenylacet-aldehyde
3,4-Dihydroxy-mandelaldehyde
4-Hydroxy-phenylethanol
Salidroside
4-Hydroxy-phenylacetaldehyde
4-Hydroxy-phenylpyruvate
Hydroxy-phenyllactate
2-Hydroxy-3-(4-hydroxy-phenyl)propenoate
3-(3,4-Dihydroxy-phenyl)lactate
3-Methoxy-4-hydroxy-phenylglycol-aldehyde
3,4-Dihydroxy-phenylethyl-eneglycol
Phenylalanine metabolism
Homoprotocatechuate
4-Hydroxy-phenylacetate
Homogentisate
Gentisate-aldehyde
3-Methoxy-4-hydroxyphenyl-acetaldehyde
3,4-Dihydroxy-phenylacetate
3,4-Dihydroxy-mandelate
3-Hydroxy-phenylacetate
2-Hydroxy-5-carboxy-methylmuconate semialdehyde
4-Chloro-phenyl-acetate
4-Hydroxy-pheny-lacetyl-CoA
4-Maleyl-acetoacetate
Gentisate
Rosmarinate
Homovanillate
3-Methoxy-4-hydroxy-phenylethylene-glycol
3-Methoxy-4-hydroxy-mandelate
DDT degradation
5-Carboxymethyl-2-hydroxymuconate
3-Maleyl-pyruvate
Hydroquinone
5-Carboxy-2-oxohept-3-enedioate
4-Hydroxyphenyl-acetylglutamic acid
4-Hydroxyphenyl-acetylglycine
4-Fumaryl-acetoacetate
3-Fumaryl-pyruvate
2-Oxohept-3-enedioate
2-Hydroxyhepta-2,4-dienedioate
2,4-Dihydroxyhept-2-enedioate
HpaG
HpaH
HpaI
Fumarate
Succinate semialdehyde
Acetoacetate
Pyruvate
Succinate
Citrate cycle
Pyruvate metabolism
1.14.18.1
1.10.3.1
5.3.3.12
4.1.99.2
1.13.11.29
1.13.11.30
1.11.1.8
4.1.1.25
4.1.1.28
1.14.16.2
1.14.17.1
2.1.1.28
2.1.1.6
2.1.1.27
2.1.1.-
2.6.1.1
2.6.1.5
2.6.1.9
2.6.1.57
2.6.1.58
1.4.1.20
4.3.1.23
5.4.3.6
2.6.1.49
1.13.12.15
4.3.1.22
1.4.3.4
1.4.3.21
1.4.9.2
1.4.3.2
1.1.1.222
1.1.1.237
1.1.1.90
4.1.1.80
1.2.1.53
5.3.2.1
1.2.1.5
1.2.1.29
1.2.3.13
1.13.11.27
1.1.1.1
1.14.14.9
1.14.13.18
4.1.1.-
1.13.12.-
1.13.11.15
1.14.12.9
6.2.1.-
1.13.11.5
1.2.3.1
2.3.1.140
1.2.1.60
1.1.1.312
1.13.11.4
4.1.1.62
5.2.1.2
5.2.1.4
2.3.1.14
2.3.1.-
5.3.3.10
4.1.1.68
3.7.1.2
3.7.1.5
3.7.1.20
1.2.1.16
00350 1/6/15
(c) Kanehisa Laboratories
Up-regulated, significant
Up-regulated, non-significant
Down-regulated, significant
Down-regulated, non-significant

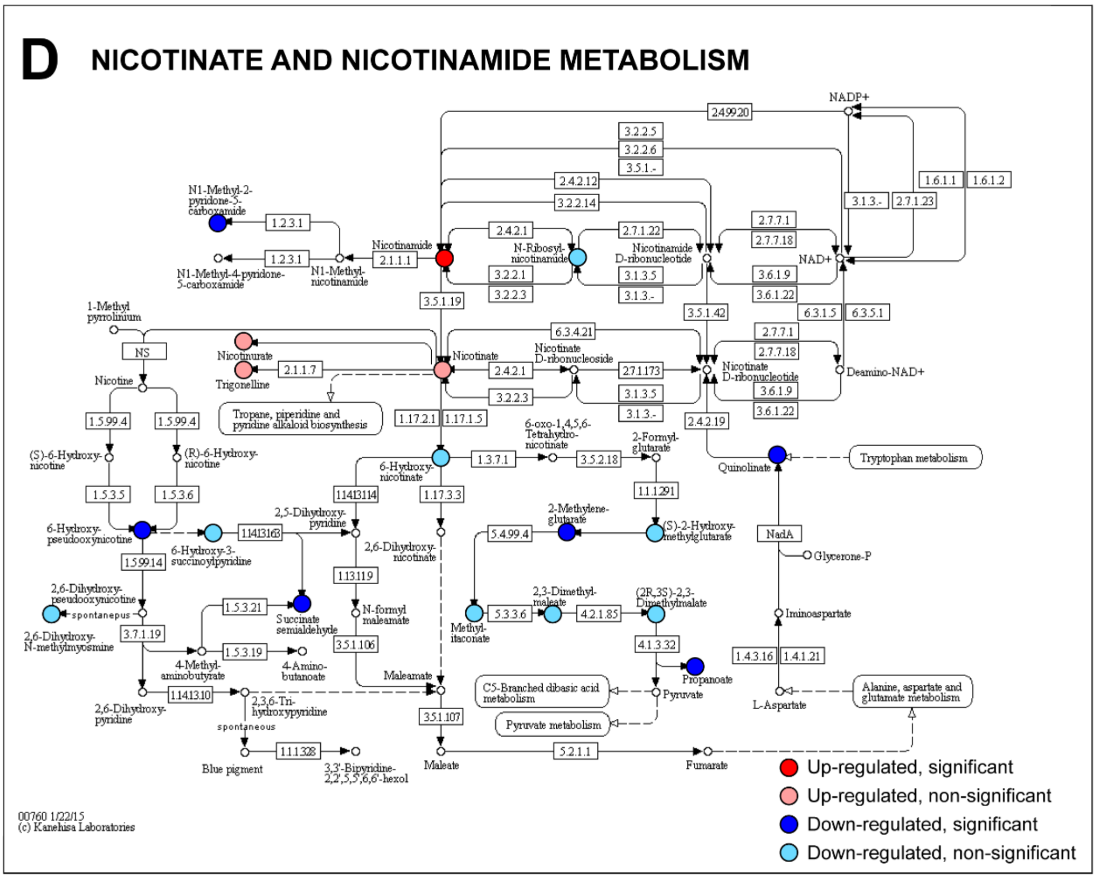

Figure S3. The complete KEGG pathway maps of the potentially regulated metabolic pathways. A: the lysine degradation pathway; **B**: the arginine and proline metabolism pathway; **C**: the tyrosine metabolism pathway; **D**: the nicotinate and nicotinamide metabolism pathway. Identified metabolites with direction of changes are indicated with different colored circles.

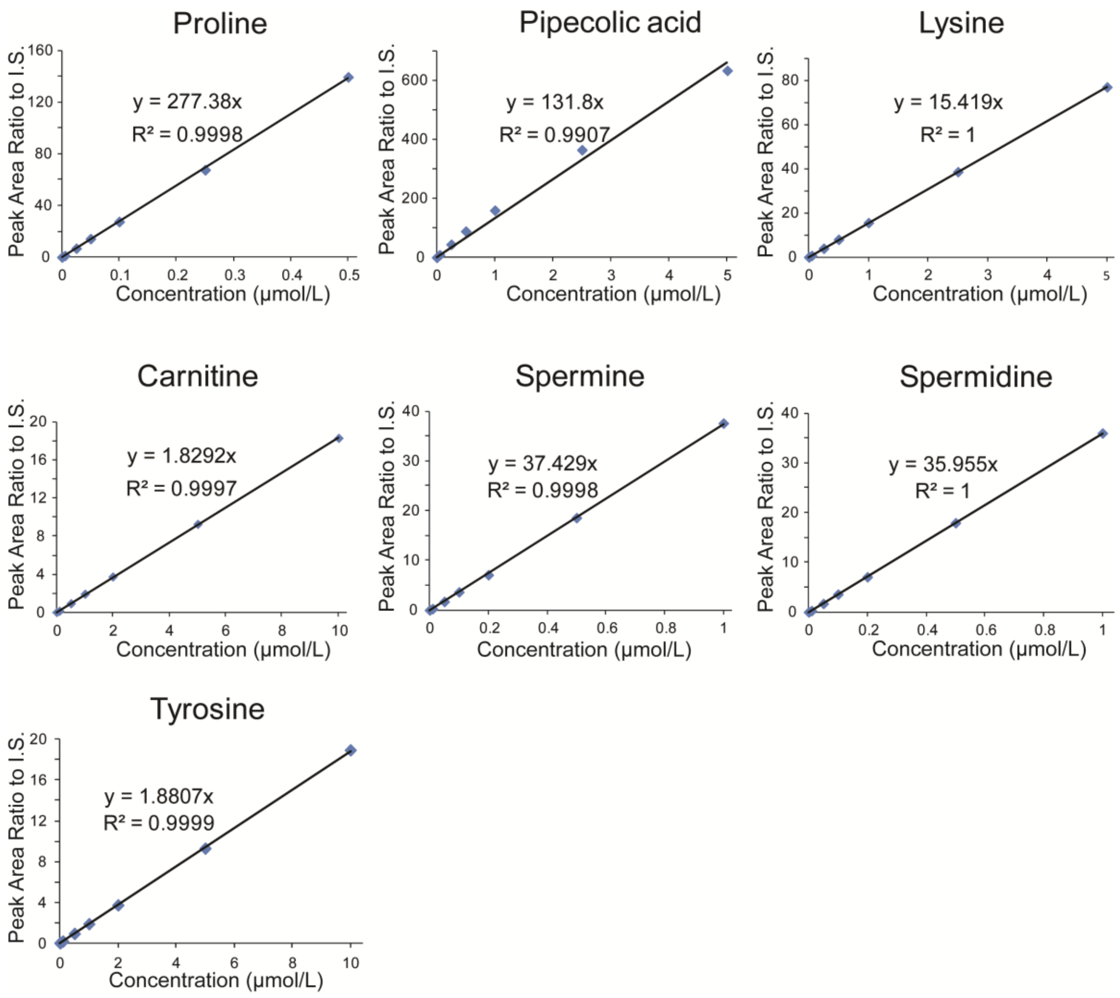

Figure S4. Calibration curves for absolute quantification of selected metabolites. Eight-point calibration curves were constructed with increasing amounts of each metabolite and a fixed concentration of the corresponding isotope-labeled internal standard (I.S.).

American Urological Association Symptom Question 2, Frequency:

Over the past month, how often have you had to urinate again less than 2 hours after you finished urination? (Not at all=0, Less than1 time in 5=1, Less than half the time=2, About half the time=3, More than half the time=4, Almost always=5)

American Urological Association Symptom Question 4, Urgency:

Over the past month, how often have you found it difficult to postpone urination? (Not at all=0, Less than1 time in 5=1, Less than half the time=2, About half the time=3, More than half the time=4, Almost always=5)

Patient/Control Exclusion Criteria:

- **Disease Exclusion:**
  - Diabetes mellitu
  - Neurologic disease
  - Chronic prostatitis
  - Chronic renal insufficiency
  - Urinary tract infection within the past 2 months
- **Clinical Chemistry Exclusion:**
  - Proteinuria >1+
  - Hematuria >2 red blood cells per high power field on urinalysis
  - Pyuria >5 white blood cells per high power field on urinalysis
  - Serum Creatinine > 1.4
- **Medical History Exclusions:**
  - Urinary tract instrumentation within the preceding 14 days
  - Prior surgical or minimally invasive therapy for benign prostatic hyperplasia
  - Active sacral nerve stimulation or equivalent
- **Drug Exclusions:**
  - Anticholinergic
  - Finasteride
  - Dutasteride
  - Complimentary alternative medicines for benign prostatic hyperplasia (eg. saw palmetto)

Figure S5. Inclusion and exclusion parameters for patient recruitment. AUA symptom index is a standard method to evaluate LUTS in men.

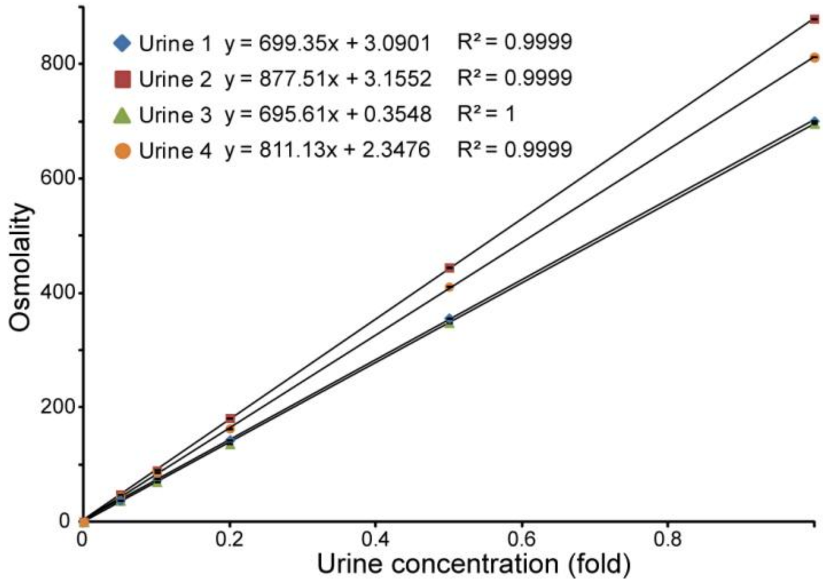

Figure S6. Osmolality linearity of human urine metabolite fractions. Four randomly selected urine samples were serially diluted to 2, 5, 10, and 20 folds, and their osmolality values were measured twice. The excellent osmolality linearity demonstrated the applicability of osmolality normalization for urine samples. Additionally, the osmolality of urinary metabolite fractions were not significantly different between disease (722±356 Osmoles/kg H_2O) and control groups (807±294 Osmoles/kg H_2O) (*p*-value = 0.37), suggesting that the total urinary metabolite output is not influenced by this disease.

Table S1. Potentially regulated metabolic pathways.

Metabolic Pathway	KEGG ID	# identified metabolites	*p*-value[a]
Lysine degradation	Map00310	16	0.02
Arginine and proline metabolism	Map00330	31	0.04
Nicotinate and nicotinamide metabolism	Map00706	18	0.08
Tyrosine metabolism	Map00350	33	0.08

[a] The pathway's *p*-value was calculated as the median of *p*-values of all the identified metabolites involved.

Table S2. Clinical information of recruited subjects.

(A) Subjects for metabolomics analysis

	LUTS	Control	*p*-value[a]
Sample size	26	20	—
Sex	Male	Male	—
Age	66 ± 11	59 ± 14	0.07
Body mass Index (BMI)	29.6 ± 5.5	31.2 ± 6.8	0.21
(B) Subjects for collagen staining analysis			
	LUTS	Control	*p*-value[a]
Sample size	5	7	—
Sex	Male	Male	—
Age	63 ± 9	60 ± 5	0.46
Tangent modulus[b]	1978 ± 314 kPa	411 ± 274 kPa	0.00002

[a] Two-tailed *t*-test. Age and BMI are not significantly different between LUTS and Control groups

[b] Tangent modulus represents the passive stiffness of periurethral tissue of the patient in kPa. The periurethral tissues of LUTS patients have significantly higher tangent modulus values than the ones of control patients, corresponding to greater tissue stiffness in LUTS.

Table S3. A list of 63 identified metabolites of interest.

Compound	m/z	Δppm[c]	Time	Ratio[d]	*p*-value[e]	*q*-value[f]
Tyrosine[a]	182.0813	0.6	1.6	-1.4	7.6E-5	9.7E-4
Spermidine[a]	146.1652	0.0	0.6	1.6	8.9E-3	1.6E-2
spermine[a]	203.2232	0.8	0.7	2.5	7.3E-3	1.4E-2
Carnitine[a]	162.1125	0.0	0.9	-2.4	1.9E-5	4.5E-4
Pipecolic acid[a]	130.0865	1.7	1.2	2.0	1.0E-2	1.6E-2
N-Acetyl-glutamate[a]	190.0710	0.1	1.8	-1.6	2.2E-8	2.8E-6
Methyloxovaleric acid[a]	131.0704	1.6	10.7	-17.5	1.3E-3	5.0E-3
N6,N6-Dimethyl-lysine[a]	175.1440	0.2	0.8	1.3	2.3E-2	2.5E-2
Citrulline[a]	176.1029	0.0	0.9	-1.7	1.2E-5	3.6E-4
Vanillylamin[b]	154.0863	0.3	2.7	2.1	3.5E-3	8.8E-3
Asp-Phe[b]	281.1132	0.1	8.8	1.5	3.8E-3	9.3E-3
Thiamine phosphate[b]	346.0856	0.9	13.6	11.2	7.8E-4	3.9E-3
Indolepyruvic acid (+NH4)[b]	221.0896	0.0	8.2	2.5	3.6E-6	1.6E-4
N-Methylmescaline[b]	226.1437	0.0	17.2	-5.4	6.4E-4	3.4E-3
2-Octenedioic acid[b]	173.0809	0.2	12.7	-1.6	3.4E-3	8.7E-3
6-Hydroxypseudooxynicotine[b]	195.1129	0.5	12.5	-1.6	1.5E-5	4.1E-4
S-Allylcysteine[b]	162.0584	0.3	9.0	3.2	5.0E-3	1.1E-2
Ketoleucine[b]	131.0704	1.6	6.5	-7.8	3.3E-4	2.3E-3
Hydroxy-pentahydroxyhexylamino-butyric acid[b]	284.1343	1.1	12.1	-5.0	2.0E-2	2.4E-2
Methylquinoline[b]	144.0809	0.7	10.0	-1.9	1.1E-7	1.0E-5
Dimethyltryptamine(+NH4)	206.1651	0.1	11.4	5.0	2.4E-7	2.0E-5
4-(Ethylamino)-4-oxo-2-butenoic acid	144.0656	1.1	4.1	1.9	1.5E-2	2.1E-2
Dihydroxyadenine (+NH4)	185.0785	1.9	4.8	3.1	9.3E-8	9.9E-6
Arborinine	286.1082	2.8	15.7	3.1	1.4E-2	2.0E-2
1-(Ethylcarbamoyl)isonipecotic acid (+Na)	223.1053	0.1	6.0	4.2	8.4E-9	2.0E-6
Oxohexanoic acid	131.0704	1.6	5.1	-8.1	2.3E-4	1.9E-3
N6-Methyl-2-deoxyadenosine	266.1236	4.5	15.1	2.1	1.4E-2	2.0E-2
Volkenin	288.1078	0.1	10.2	-3.5	4.8E-4	3.0E-3
Dopaxanthin quinone	389.0955	4.8	11.3	10.3	1.9E-2	2.4E-2
Dichloro-oxoadipate	226.9514	2.1	0.7	-2.3	6.3E-5	8.8E-4
Deaza-adenosine	267.1075	4.5	12.1	-8.6	8.0E-3	1.5E-2
Pyridosine	255.1339	0.2	2.6	10.0	2.8E-3	8.0E-3
Euchrenone b1	475.2476	0.6	18.0	2.4	1.2E-8	2.0E-6
Methylglutaric acid	147.0652	0.1	0.8	-1.7	2.9E-4	2.1E-3
N-Heptanoylhomoserine lactone	214.1438	0.1	16.7	9.9	3.6E-4	2.4E-3
2-(4-Hydroxyphenyl)propionate	166.0626	0.9	14.3	1.5	4.2E-2	3.5E-2
Diethyl-diethoxy-phenylene-biscarbamate	341.1708	0.3	14.1	1.3	4.3E-2	3.5E-2
Diethyl oxalpropionate	203.0914	0.4	15.1	1.9	2.7E-2	2.8E-2
N-Benzyloxycarbonyl-leucine	266.1387	0.1	12.9	1.4	5.8E-3	1.2E-2
Retronecine	156.1019	0.5	1.5	-1.9	1.9E-3	6.4E-3
Ilicifolinoside A	265.1284	0.8	13.8	-8.0	1.1E-2	1.7E-2
4-Methyldibenzothiophene	199.0578	0.9	8.5	5.9	3.3E-3	8.7E-3
(1-Methylpentyl)succinate	201.1122	0.2	13.6	1.7	3.0E-3	8.3E-3
Indole carboxyl tetrahydro-thiazole	249.0694	0.7	17.1	-12.0	5.0E-2	3.8E-2
Citrylglutamate	322.0769	0.0	1.7	-1.4	2.5E-5	5.1E-4
Iridodial	169.1224	0.4	16.3	-1.6	3.5E-3	8.8E-3

2-Amino-diethoxy-phosphoryloxy-propionic acid	242.0788	0.1	18.0	2.8	4.5E-4	2.9E-3
4-Nitropheylhydrazine	154.0611	0.2	1.7	-1.3	5.9E-3	1.2E-2
Hydroxytyrosol	155.0703	0.0	12.7	-1.6	1.5E-2	2.1E-2
Pseudopelletierine	154.1227	0.2	18.0	10.1	8.4E-5	1.0E-3
5-Hydroxyferulate	211.0600	0.1	13.0	-1.8	1.9E-2	2.3E-2
Furcatin	429.1734	4.9	17.3	1.6	2.5E-3	7.5E-3
Triphenyl-(2-phenylethyl)-pyrrole-2-carboxamide	443.2128	2.4	17.1	-7.1	1.1E-2	1.8E-2
2-Diphenylphosphanylbenzoate	561.3124	1.1	18.1	-6.7	2.6E-2	2.7E-2
Oxyapramycin (+Na)	578.2656	2.1	16.1	-5.7	1.6E-2	2.1E-2
Bis-hexylthio-thioisonicotinamide	371.1661	4.8	13.9	-4.7	2.9E-2	2.9E-2
Liensinine	575.2915	0.3	18.2	-5.8	2.9E-2	2.9E-2
N-Acetylpuromycin (+NH_4)	531.2655	3.6	17.9	-8.0	1.8E-2	2.3E-2
L-Aminoadipate adenylate (+NH_4)	509.1635	1.1	17.3	1.4	2.6E-3	7.7E-3
Trityloxy-hexyl-oxyphthalonitrile	487.2387	1.4	17.6	-7.4	1.5E-2	2.0E-2
Acetamidobenzoyl-aminohydroxybutanoic acid	281.1133	0.4	7.5	-1.6	9.2E-3	1.6E-2
Dethiobiotin	215.1391	0.4	4.5	1.4	3.9E-4	2.6E-3
3-Hydroxycotinine glucuronide	369.1294	0.4	13.9	-1.7	6.5E-5	8.8E-4

[a] Confirmed with metabolite standard compounds.

[b] Confirmed with MS/MS fragmentation.

[c] Δppm mass error = $1\times10^6\times$|detected m/z – theoretical m/z| / theoretical m/z.

[d] Ratio>0 : up-regulated metabolite; Ratio<0 : down-regulated metabolite.

[e] *p*-value was calculated from Student's *t*-test.

[f] *q*-value was calculated from FDR method.
